# Chaperone dysfunction in motor neuron disease: new insights from studies of the SMN complex

**DOI:** 10.1093/genetics/iyae223

**Published:** 2025-02-05

**Authors:** A Gregory Matera

**Affiliations:** Integrative Program for Biological and Genome Sciences, University of North Carolina School of Medicine, University of North Carolina at Chapel Hill, Chapel Hill, NC 25799, USA; Departments of Biology and Genetics, University of North Carolina at Chapel Hill, Chapel Hill, NC 27599, USA; RNA Discovery Center and Lineberger Comprehensive Cancer Center, University of North Carolina School of Medicine, University of North Carolina at Chapel Hill, Chapel Hill, NC 27599, USA

**Keywords:** heat shock proteins, hspA, ALS, amyotrophic lateral sclerosis, SMA, spinal muscular atrophy, survival motor neuron, SMN, TDP-43, genetic suppressors

## Abstract

Spinal muscular atrophy and amyotrophic lateral sclerosis are devastating neurodegenerative diseases characterized by motor neuron loss. Although these 2 disorders have distinct genetic origins, recent studies suggest that they share common etiological mechanisms rooted in proteostatic dysfunction. At the heart of this emerging understanding is the survival motor neuron (SMN) complex.

The survival motor neuron (SMN) complex is traditionally known for its central role in the biogenesis of small nuclear ribonucleoproteins (snRNPs), as reviewed in Matera and Wang (2014) and Gruss *et al*. (2017). Composed of SMN and its Gemin protein partners, the complex has long been recognized as an assembly chaperone for RNPs (Chari *et al*. 2008). Mutations in the human *SMN1* gene cause SMA, leading to reduced levels of functional SMN protein. However, the role of the SMN complex extends beyond its canonical function in spliceosomal snRNP assembly (Praveen *et al*. 2012). Studies over the past dozen years or so have shown that its noncanonical roles intersect with other crucial cellular processes including mRNP transport, mRNA translation, endocytosis, neuritogenesis, autophagy, protein homeostasis, cytoskeletal maintenance, and innate immune signaling (Navascues *et al*. 2004; Fallini *et al*. 2016; Hosseinibarkooie *et al*. 2016; Deguise *et al*. 2017; Donlin-Asp *et al*. 2017; Singh *et al*. 2017; Chaytow *et al*. 2018; Massenet 2019; Garcia *et al*. 2024). Importantly, each of these processes is likely to require the activity of molecular (folding) chaperones.

*Quis custodiet ipsos custodes?*

This perspective article brings us to a fundamental question in cellular biology, elegantly captured by the Latin phrase, quoted above, meaning—Who guards the guards? Or in this case—Who chaperones the chaperones? As we delve into several recent discoveries, we'll explore how disturbances in the proteostasis network can upset the delicate balance between molecular chaperones and their various clients. In particular, we will discuss the hypothesis that perturbations involving molecular chaperones lie at the etiological root of both SMA and ALS.

## Heat shock proteins and spinal muscular atrophy

Heat shock proteins (HSPs) comprise a large and highly-conserved family of molecular (folding) chaperones (Lindquist and Craig 1988; Kampinga *et al*. 2009). Members of the HspA/Hsc70 subfamily, many of which are constitutively expressed, play essential roles in preventing protein aggregation, assisting in the refolding of stress-denatured proteins, and facilitating the degradation of irreparably damaged ones (Hartl *et al*. 2011; Miles *et al*. 2019). In the context of motor neurons, which are particularly vulnerable to proteostatic stress (Brehme *et al*. 2014; Calderwood and Murshid 2017), heat shock proteins are essential not only for preventing neurodegeneration but also for proper synaptic vesicle priming and recycling (see below).

**Fig. 1.**
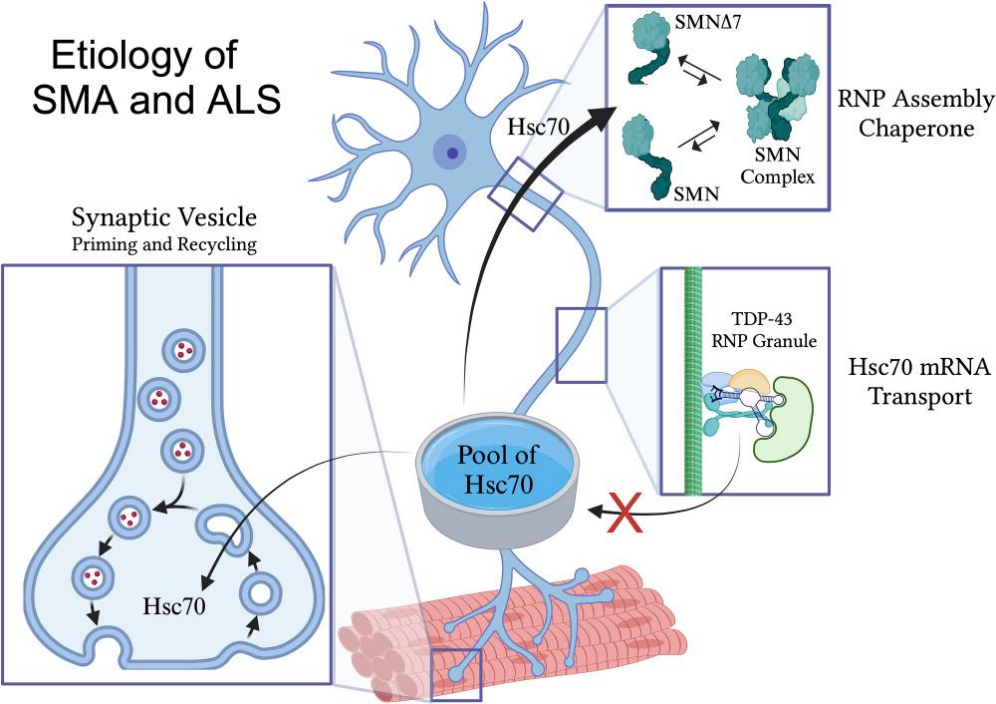
Changes in the available pool of the heat shock cognate protein Hsc70-4/HspA8 (Hsc70) at motor axon terminals may be ultimately responsible for defects in synaptic vesicle priming and recycling, two hallmarks of motor neuron disease. As outlined in the text, the SMN complex is itself an assembly chaperone for RNPs. Misfolding or truncation of its eponymous component, SMN protein, can lead to defects in formation of the complex, thereby redirecting the available pool of Hsc70 folding chaperone away from its normal synaptic vesicle clients and leading to SMA. Alternatively, failure of Hsc70 mRNA transport would result in an overall decrease in the size of the pool of Hsc70 protein, thus leading to ALS. See text for additional details. SMA, spinal muscular atrophy; ALS, amyotrophic lateral sclerosis; SMN, full-length survival motor neuron; SMNΔ7, truncated form of SMN lacking exon 7 sequences; TDP-43, TAR DNA-binding protein-43. Created using Biorender.com.

Recent work in animal models of SMA has uncovered a link between SMN and Hsc70-4. In *Drosophila*, affinity purification coupled with mass spectrometry (AP-MS) revealed that Hsc70-4 and other HspA family members preferentially associate with mis-folded, SMA-causing alleles of SMN (Matera *et al*. 2024). This finding is especially intriguing when viewed in light of a groundbreaking study in mice. Monani and colleagues identified a missense mutation in HspA8 (the mammalian ortholog of Hsc70-4) that acts as a potent suppressor of the SMA phenotype, bypassing the need for high levels of full-length SMN (Kim *et al*. 2023). The suppressor mutation (G470R) is located in the substrate recognition domain of HspA8/Hsc70-4, and it reduces its binding affinity for SMN (Kim *et al*. 2023). The authors propose that the reduced affinity allows the cell to “redirect” this folding chaperone away from aberrantly formed SMN complexes present in SMA mice and onto other crucial Hsc70 clients that are required for proper neurotransmission (Fig. 1). The subsequent *Drosophila* studies elaborate upon this idea and provide additional evidence for the critical role of heat shock chaperones in the context of motor neuron disease.

Interestingly, the suppressive effect of the HspA8 point mutation depends on the presence of transgenic copies of *SMNΔ7* in the background of the SMA model mice (Kim *et al*. 2023). SMNΔ7 is the principal protein product of the *SMN2* gene and is lacking key YG-box residues encoded by exon 7 (Lorson *et al*. 1998; Le *et al*. 2005). As illustrated in Fig. 1, this truncated isoform is primarily monomeric (Gupta *et al*. 2021). Moreover, it fails to homo-dimerize, although it is able to form heterodimers with full-length SMN (Lorson *et al*. 1998; Gupta *et al*. 2015; Gupta *et al*. 2021). The SMNΔ7 truncation exposes an F-box phosphodegron that preferentially targets this isoform for proteasomal degradation in both flies and humans (Gray *et al*. 2018). In human cells, overexpression of SMNΔ7 has a protective effect on full-length SMN levels (Gray *et al*. 2018). In SMA model mice, the *SMNΔ7* transgenes likely provide an extra measure of SMN protein activity (Le *et al*. 2005). However, in the context of human SMA, the relative inability of the SMNΔ7 promote proper formation of the oligomeric SMN complex may also represent a proteostatic stress signal that calls for an Hsc70 response (Fig. 1).

## Molecular chaperones are required for normal synaptic function

Heat shock proteins play a central role in neurotransmission as both gatekeepers and reclamation factors, as reviewed in Gorenberg and Chandra (2017). Together with its co-chaperone Csp⍺ (a.k.a. DnaJc5, a member of the Hsp40/DNAJ subfamily of HSPs), Hsc70 is required for the proper priming and recycling of synaptic vesicles (SVs). In most cell types, exocytic vesicle targeting and fusion is carried out by SNAPs (soluble NSF attachment proteins) and SNAREs (SNAP receptors). In neurons, the SNAPs become misfolded after each cycle of SV fusion and must be refolded into a SNARE assembly-competent conformation before they can be used again (Sharma *et al*. 2011). This process is called SV priming (Fig. 1), and it requires the ATP-dependent chaperone activity of Hsc70 and Csp⍺ in order to generate assembly-competent conformers of a protein called Snap25 (Sollner *et al*. 1993; Whiteheart *et al*. 1993). Notably, the incompetent Snap25 conformers are targeted for destruction by the proteosome (Sharma *et al*. 2011); so, Hsc70 is essential for activity-dependent neurotransmitter release (Uytterhoeven *et al*. 2015). In addition, Hsc70 independently functions in several key steps of endocytic recycling (Fig. 1), including the process of clathrin-uncoating (Eisenberg and Greene 2007; Uytterhoeven *et al*. 2015; Parra *et al*. 2016). Thus, Hsc70 can be viewed as both a gatekeeper of vesicle fusion and as a recycler of synaptic membranes. Both of these pathways are required to maintain proper neurotransmission.

Importantly, synaptic function also depends on localized translation of Hsc70 folding chaperones at, or near, axon terminals (Alecki *et al.* 2024). Localized protein synthesis requires active mRNP transport down the axon, a process that is mediated by RNA-binding proteins (RBPs) and other transport factors. This is where the story gets really interesting, because mutations in a number of genes that encode RBPs like TDP-43, FUS, and C9orf72 are known to cause ALS. Mutations in these proteins are often dominant negative and characterized by the presence of RNP-rich inclusions or condensates. One such mutation in TDP-43 causes the aberrant sequestration of the mRNA that encodes—you guessed it—Hsc70-4/HspA8 (Coyne *et al*. 2017). Crucially, overexpression of Hsc70 can suppress the phenotypes in both mouse and *Drosophila* ALS disease models (Coyne *et al*. 2017).

As outlined in Fig. 1, these findings show that limiting the available pool of Hsc70 chaperones on the supply side of the equation can have similar detrimental effects on neuromuscular function. Thus, reduced levels of Hsc70-4/HspA8 expression in axons and dendrites, observed across multiple models (Coyne *et al*. 2017; Alecki *et al.* 2024), is coupled with an impairment of synaptic vesicle endocytosis (Fig. 1). Together with other recent studies on TDP-43 and C9orf72 models (Coyne *et al*. 2017; Chitiprolu *et al*. 2018), the new work on SMN (Kim *et al*. 2023) suggests that SV cycling defects may be a common pathomechanism in ALS and related disorders.

## The SMN complex: beyond RNP assembly

Although the SMN complex's role in snRNP assembly is well-established, numerous studies have revealed a much broader network of physical interactions (Trinkle-Mulcahy *et al*. 2008; Chaytow *et al*. 2018; Matera *et al*. 2019; Matera *et al*. 2024), extending our understanding of the complex's functions beyond its canonical role. In *Drosophila*, these interactions include components of the nuclear cap–binding complex, the signal recognition particle, and various ATPases. Particularly noteworthy to the topic at hand is the recent finding (Matera *et al*. 2024) that SMN co-purifies with 3 other components of the endocytic recycling machinery: synapsin, DAP160, and Snap29 (Winther *et al*. 2015; Mastrodonato *et al*. 2018; Hoffmann *et al*. 2023).

How SMN might be involved (directly or indirectly) in recycling of SVs is currently unclear. There are numerous genetic and physical interactions reported in the literature between the SMN complex and components of the autophagic and innate immune signaling networks, see study by Garcia *et al*. (2024) and references therein. These diverse interactions are underscored by the concept of “emergent” properties of cellular networks. An emergent property is one that develops as a consequence of the various interactions within a network that cannot be elucidated from the properties of its individual elements (Nardai *et al*. 2006). Thus, chaperone-related immune dysfunction may be an emergent property of a distorted proteostasis network. In this context, the effects of mutations in SMN or its interacting partners cannot be understood in isolation but must be considered within the broader web of cellular interactions. This view is particularly relevant when interpreting the phenotypes of gain-of-function and loss-of-function mutations in the context of the proteostasis network.

For instance, the ALS-causing mutant of TDP-43 that sequesters Hsc70-4 mRNA could be viewed as a gain-of-function mutation (Coyne *et al*. 2017). However, its ultimate effect is to reduce the availability of a crucial chaperone, resembling a loss-of-function scenario. Similarly, the HspA8^G470R^ mutation that suppresses SMA phenotypes is technically a partial loss-of-function mutation, but its net effect is beneficial in the context of reduced SMN levels (Kim *et al*. 2023). Genetic suppression is a complicated process even when dealing with isogenic strains. In humans, polymorphic variants (e.g. in chaperones or other assembly factors) are likely to distort their associated signaling networks in unpredictable ways that could either suppress or induce disease states. The examples noted above illustrate how the traditional binary classification of mutations can become muddled when considering their effects within complex cellular networks.

Despite the fact that ALS and SMA have a very similar phenotypic presentation in humans, common genes and pathways have not previously been identified. It is curious to note that in the original purification of SMN-binding partners from HeLa cells, Hsc70 was identified as a potential component of the complex (Meister *et al*. 2001). However, without any functional evidence tying heat shock chaperones to an organismal phenotype, Hsc70 was dismissed as a contaminant. Although it may not be surprising to the readership of this journal, the importance of animal models and genetic suppression analysis in elucidating a functional connection between HspA8/Hsc70-4 and SMN cannot be overstated.

## Implications and future directions

In summary, recent findings paint a picture of the SMN complex as a multifunctional hub, integrating various cellular processes including RNP assembly, proteostasis, and innate immunity (Garcia *et al*. 2024; Matera *et al*. 2024). This expanded view of SMN function provides a new perspective regarding the molecular mechanisms underlying SMA and potentially other motor neuron diseases, opening up novel avenues for therapeutic intervention. The emerging picture of chaperone dysfunction in motor neuron disease challenges our understanding of SMA and ALS etiology. Rather than viewing these diseases solely through the lens of RNP assembly defects or protein aggregation, we must consider them as complex disturbances in the proteostasis network, with repercussive impacts on synaptic function.

These insights open up new avenues for therapeutic intervention. Although current SMA treatments have focused on increasing SMN levels via splice-altering drugs, the new findings suggest that modulating the activity of molecular chaperones like Hsc70-4/HspA8 could be a promising alternative or combinatorial approach. This could involve developing small molecules that enhance chaperone function or increase their expression at synaptic terminals. Another avenue would be to identify bioactive compounds that allow SMNΔ7 to form oligomers together with full-length SMN. With regard to ALS, the observation that overexpression of Hsc70-4, its co-chaperone Csp⍺ or their client, dynamin, can rescue SV defects in TDP-43 models (Coyne *et al*. 2017) suggests that these proteins and their interactions could be potential therapeutic targets. Developing strategies to enhance the function of the Csp⍺/Hsc70 chaperone complex, or its clients involved in SV cycling, could help mitigate synaptic dysfunction in ALS and related disorders (Burgoyne and Morgan 2015; Imler *et al*. 2019). Given the role of mutant TDP-43 in sequestering Hsc70-4 mRNPs, developing RNA-based therapies that prevent this sequestration or enhance translation of the mRNA could be a novel approach to maintaining synaptic health in ALS. The complex nature of chaperone dysfunction in motor neuron diseases suggests that combination therapies targeting multiple aspects of proteostasis and synaptic function may be more effective than single-target approaches (Groen *et al*. 2018; Wirth *et al*. 2020; Antonaci *et al*. 2023).

As we move forward, several key questions remain to be addressed in future research. We need to better understand how mutations or changes in SMN levels affect the global landscape of protein–protein interactions in neurons, particularly those involving chaperone proteins. The precise mechanisms by which TDP-43 and C9orf72 repeat expansions regulate Hsc70 expression and synaptic vesicle cycling also require further elucidation. Additionally, identifying other key chaperone proteins or co-chaperones involved in maintaining synaptic health in motor neurons, and understanding how they interact with the SMN complex and TDP-43, will be crucial for developing comprehensive therapeutic strategies.

The development of biomarkers based on synaptic vesicle cycling defects or chaperone dysfunction could enable earlier diagnosis or help monitor disease progression in ALS and SMA. This could be particularly valuable for assessing the efficacy of potential treatments in clinical trials. Furthermore, understanding how age-related changes in the proteostasis network contribute to the onset and progression of motor neuron diseases may lead to interventions that can delay these age-related effects.

The role of the innate immune system in the progression of SMA and ALS, and its relationship to molecular chaperones, is another area that warrants further investigation (Deguise and Kothary 2017; Garcia *et al*. 2024; Matera *et al*. 2024). Recent studies have hinted at connections between innate immunity and proteostasis (Brehme *et al*. 2014; Yang *et al*. 2024), suggesting that this interaction could be a fruitful avenue for therapeutic development. As we continue to uncover commonalities in synaptic dysfunction across different neurodegenerative disorders (e.g. ALS, SMA, Alzheimer's disease, and Parkinson's disease), we may identify shared pathways that could be targeted therapeutically. This broader perspective could lead to the development of treatments with potential applications beyond motor neuron diseases.
